# Evaluation of a treatment protocol for anaemia in pregnancy nested in routine antenatal care in a limited-resource setting

**DOI:** 10.1080/16549716.2019.1621589

**Published:** 2019-06-17

**Authors:** Mary Ellen Gilder, Julie A. Simpson, Germana Bancone, Laura McFarlane, Neha Shah, Rob van Aalsburg, Moo Koh Paw, Mupawjay Pimanpanarak, Jacher Wiladphaingern, Aung Myat Min, Claudia Turner, Marcus J. Rijken, Machteld Boel, Gabie Hoogenboom, Nay Win Tun, Prakaykaew Charunwatthana, Verena I. Carrara, François Nosten, Rose McGready

**Affiliations:** a Shoklo Malaria Research Unit, Mahidol-Oxford Tropical Medicine Research Unit, Faculty of Tropical Medicine, Mahidol University, Mae Sot, Thailand; b Centre for Epidemiology and Biostatistics, Melbourne School of Population and Global Health, The University of Melbourne, Melbourne, Australia; c Centre for Tropical Medicine and Global Health, Nuffield Department of Medicine, University of Oxford, Oxford, UK; d Cambodia Oxford Medical Research Unit, Angkor Hospital for Children, Siem Reap, Cambodia; e Department of Obstetrics and Gynaecology, University Medical Centre Utrecht, Utrecht University, Utrecht, The Netherlands; f Mahidol-Oxford Tropical Medicine Research Unit, Faculty of Tropical Medicine, Mahidol University, Bangkok, Thailand; g Department of Medicine, Swiss Tropical and Public Health Institute, Basel, Switzerland

**Keywords:** Anaemia, health worker, malaria, iron deficiency, G6PD deficiency, haemoglobinopathy, Myanmar, medical error, prophylaxis, protocol

## Abstract

**Background**: Anaemia in pregnancy is typically due to iron deficiency (IDA) but remains a complex and pervasive problem, particularly in low resource settings. At clinics on the Myanmar–Thailand border, a protocol was developed to guide treatment by health workers in antenatal care (ANC).

**Objective**: To evaluate the clinical use of a protocol to treat anaemia in pregnancy.

**Methods**: The design was a descriptive retrospective analysis of antenatal data obtained during the use of a standard anaemia treatment protocol. Two consecutive haematocrits (HCT) <30% prompted a change from routine prophylaxis to treatment doses of haematinics. Endpoints were anaemia at delivery (most recent HCT before delivery <30%) and timeliness of treatment initiation. Women whose HCT failed to respond to the treatment were investigated.

**Results**: From August 2007 to July 2012, a median [IQR] of five [4–11] HCT measurements per woman resulted in the treatment of anaemia in 20.7% (2,246/10,886) of pregnancies. Anaemia at delivery was present in 22.8% (511/2,246) of treated women and 1.4% (123/8,640) who remained on prophylaxis. Human error resulted in a failure to start treatment in 97 anaemic women (4.1%, denominator 2,343 (2,246 + 97)). Fluctuation of HCT around the cut-point of 30% was the major problem with the protocol accounting for half of the cases where treatment was delayed greater than 4 weeks. Delay in treatment was associated with a 1.5 fold higher odds of anaemia at delivery (95% CI 1.18, 1.97).

**Conclusion**: There was high compliance to the protocol by the health workers. An important outcome of this evaluation was that the clinical definition of anaemia was changed to diminish missed opportunities for initiating treatment. Reduction of anaemia in pregnancy requires early ANC attendance, prompt treatment at the first HCT <30%, and support for health workers.

## Background

The global burden of maternal morbidity and mortality attributable to anaemia remains high in low-resource settings. A 2011 review estimated that approximately 40% of pregnant women have anaemia worldwide, with the vast majority of anaemic women (90%) residing in Africa or Asia [1]. Anaemia is the second highest cause of maternal mortality in Asia [2,3]. A fundamental goal of all targeted antenatal care (ANC) programs is to detect and correct iron deficiency anaemia (IDA) to reduce serious complications [4], and provide prophylactic iron supplementation [5]. As blood loss at delivery is expected, anaemia at delivery is particularly hazardous for women and is a marker of the success or failure of ANC interventions to treat or prevent anaemia. Studies of IDA in pregnancy have demonstrated lifelong negative health consequences for infants including poor pregnancy outcomes such as small for gestational age and preterm delivery, and later effects on educational performance, work capacity and productivity [6]. The published literature suggests that most women with anaemia living in rural, limited-resource settings where screening for IDA is cost prohibitive (and complicated by infections such as malaria) have a component of IDA and will respond to haematinics [7]. Thailand reduced the prevalence of anaemia in pregnancy from 25% in 1988 to 15% in 1997, primarily by integrating iron supplementation into primary care programs [8].

At health-care facilities serving refugees and migrant workers on the Myanmar–Thailand border anaemia is commonly diagnosed using haematocrit (HCT) and defined as HCT <30%. Though mean HCT values normally change throughout pregnancy, with a nadir in the second trimester and recovery as women approach term, this uniform threshold has been used for over 30 years to improve consistency of diagnosis by staff with varying levels of training [9]. At the Shoklo Malaria Research Unit (SMRU) clinics, 37.5% of women presenting for ANC were anaemic according to this definition [10], and anaemia at first visit was associated with a two-fold increase in maternal mortality [11].

In pregnant women presenting to SMRU clinics and in other rural Asian populations anaemia is likely to be multifactorial as iron, folic acid and B12 deficiency, helminth infections, and malaria are all prevalent [10]. Haemoglobinopathies and glucose-6-phosphate dehydrogenase (G6PD) deficiency also contribute to anaemia in a proportion of the population [12]. Resources to determine the exact cause of anaemia are often inaccessible and too costly for limited-resource settings, but clinical protocols have been shown to be highly effective to improve the delivery of appropriate clinical interventions for maternal health in these settings [13].

The protocol (Supplementary file 1) used here was developed and implemented by SMRU in 2007 following a small pilot study that showed 45% (36/80) of anaemic women were iron deficient and 33% (27/81) had a haemoglobinopathy. The aim of the protocol was to guide ANC staff in prescribing systematic haematinics and investigating women who failed to respond to treatment, in an effort to overcome the operational challenges of effective iron supplementation [14]. The objective of this service review was to determine the strengths and weaknesses of the protocol in order to improve treatment provision and pregnancy outcomes. The specific endpoints and outcomes evaluated in the service review were: timeliness of initiation of treatment in relation to anaemia diagnosis, anaemia at delivery, and relative contribution of the most common anaemia aetiologies among the cases who did not respond to standard haematinics.

## Methods

### Study design and participants

This is a retrospective analysis of ANC records during the time frame when the anaemia protocol was operational, including women enrolled in ANC after 1 August 2007 with singleton births by the end of July 2012. Women with miscarriage, multiple pregnancy (twins) or lost to follow-up before delivery were excluded. Additional exclusions were: women with less than 2 HCT values during ANC follow-up as this was the minimum number needed to meet the definition of anaemia; first ANC visit less than 4 weeks before delivery as there was no time to monitor the response; and severe acute anaemia or non-standard oral treatment because response to treatment would be atypical compared to the norm, e.g. known haemoglobinopathy from involvement in studies at SMRU [15,16].

### Setting

The research was conducted in rural clinics run by SMRU along the Myanmar–Thailand border in North Western Thailand. SMRU offers free healthcare and ANC to refugees and migrants from Myanmar covering an estimated population of 45,000 refugees (at the time of data collection) and 200,000 migrant workers. There was no selection, all SMRU clinics were included in this analysis.

### Model of ANC

The ANC model at SMRU was established in 1986, and has been described in detail [17]. Briefly, ANC was provided by health-care workers (midwives and medics) who had received training conducted by SMRU or other health-care professionals [18,19]. Doctors trained in obstetrics gave ongoing training and clinical support to staff in person during the workday and were available by phone for consultation at night or over the weekend. During the time of the review, pregnant women registered at SMRU were asked to attend appointments every 1–2 weeks for the duration of their pregnancy. This high frequency of consultations was set to provide active detection and treatment for malaria [11]. At every visit, women were asked about symptoms, and a protocol specified further procedures based on gestational age and obstetric and medical history including weight, abdominal examination, blood pressure, malaria smear, and HCT. HCT results were reported the same day and health workers used these results to make decisions about supplementation and investigations based on the protocol.

All women with positive malaria smears were treated with antimalarials, regardless of symptoms. *P.falciparum* was treated with quinine and clindamycin for 7 days in first trimester, artesunate and clindamycin for 7 days in 2nd and 3rd trimester, and *P.vivax* with chloroquine for 3 days. Pregnant women with malaria had at least a daily malaria smear done, and those who were anaemic could commence anaemia treatment when the malaria smear was negative. At the first ANC visit a dating ultrasound scan was offered, and gestational age determined preferably from: the crown rump length at 8 to <14 weeks; the head circumference from 14 to 24 weeks; and after 24 weeks the best estimate from ultrasound, Dubowitz exam or last menstrual period was determined [19].

All non-anaemic pregnant women received a prophylactic daily haematinic regimen consisting of supplemental iron (200 mg ferrous sulphate daily) and folic acid (5 mg once per week). Vitamin B1 deficiency is historically important in this population, and high levels of deficiency have resulted in a standard supplement of thiamine hydrochloride 100 mg once daily [20]. Women received their supplements in weekly packaging and were provided with the number of sachets commensurate with the number of weeks until the next ANC visit. At the first ANC visit the schedule and supplement regimen was explained to women using a teach-back method to confirm understanding. If women stated that they would be away longer than the next anticipated ANC visit, they were provided with additional sachets as needed.

### Development of the protocol and treatment of anaemia

The protocol was designed expecting most women to respond to treatment doses of iron supplementation based on the results of the pilot (Supplementary file 1). Anaemia was defined as mild (HCT 25–29%, equating to a haemoglobin (Hb) approximately 8.3 to 9.7 g/dL [21]), moderate (20–24%; Hb approximately 6.7 to <8.3 g/dL)) or severe (HCT < 20%, Hb < 6.7 g/dL). The cut-off of HCT <30% roughly correlates with the WHO definition of moderate anaemia (Haemoglobin <10.0 g/dL), but this global definition is currently being revised [22]. Treatment was initiated when anaemia was confirmed by two consecutive HCT <30% despite prophylaxis. HCT confirmation was required by the protocol to account for the natural variability of this measurement (within-subject biological variation estimated ±1.5%) [23]. For mild anaemia, the HCT was confirmed at the next visit, usually 2 weeks later, but for moderate or severe anaemia this was done as soon as possible. Anaemia at delivery was determined from the most recent measurement at or before delivery and HCT <30% was considered anaemic, and this included women with only a single low HCT, if the other inclusion criteria were met.

Anaemia treatment included iron supplementation of 200 mg ferrous sulphate (65 mg elemental iron) three times a day with 50 mg of Vitamin C at the same time, folate 5 mg once daily, and vitamin B12 100 mcg twice a day, as well as advice on the importance of compliance to the recommended daily dosing. In an attempt to improve compliance, this was changed to 400 mg ferrous sulphate twice a day in 2010. When possible, women stayed at housing for patients on the clinic property or came once or twice a day to the clinic to allow for supervised treatments, especially if the response to treatment was sub-optimal. Severe anaemia was treated with hospitalization and transfusion with screened donor blood or supervised haematinics as indicated by the clinical situation.

Adequate response to iron treatment was indicated by a 2% (0.6 g/dL) rise in HCT by the next ANC visit, usually 2 weeks later (range: 1–6 weeks), and further investigations were reserved for women whose HCT did not show this 2% increase (non-responders). Investigations included serum ferritin, a stool test, complete blood count (including mean corpuscular volume [MCV]), haemoglobin typing (for diagnosis of haemoglobinopathies) and G6PD deficiency test. IDA was defined by a serum ferritin concentration <30 ng/mL [24]. Microcytosis was defined as MCV of <80 fL.

### Laboratory

HCT was measured at the clinics on 30 µL of blood obtained via fingerstick using a heparinized capillary tube centrifuged at 10,000 rpm for 3 min, then read manually (single reading only) with a Hawksley Micro-Haematocrit reader. Malaria was confirmed by microscopy of thick and thin malaria smears, stained with Giemsa and examined under oil immersion. Smears were only declared negative after 200 fields were read. G6PD deficiency was diagnosed using the fluorescent spot test (FST, R&D Diagnostic, Greece) as described previously elsewhere [12]. Complete blood count (CBC) was performed on venous blood collected in EDTA tubes. The samples were transported refrigerated to the haematology laboratory and analysed using a Sysmex pocH-100i (Sysmex Corporation, Kobe) or CeltacF MEK-8222K haematology analyser (Nihon Kohden, Japan). Haemoglobin variants and serum ferritin levels were analysed by an external laboratory.

SMRU lab staff are involved in regular quality control and training, as well as additional quality control as required by specific studies.

### Statistical methods

ANC data, from the first visit until delivery, were analysed using SPSS for Windows version 22 (SPSS, Inc., Chicago, Illinois, USA) and Stata version 13 (StataCorp, Tx, USA). Continuous normally distributed data were described by the mean, standard deviation (SD) and non-normally distributed data by the median and inter-quartile range (IQR). Multivariable logistic regression was performed in the haematinic treatment group only, to estimate adjusted associations between the outcome anaemia at delivery and each of the following covariates: primigravida, first ANC before 3rd trimester, malaria in pregnancy, delayed treatment (started >4 weeks from diagnosis), and mean HCT at diagnosis. Only women who had received at least 2 weeks of treatment were included in the regression.

## Results

**Figure 1.**
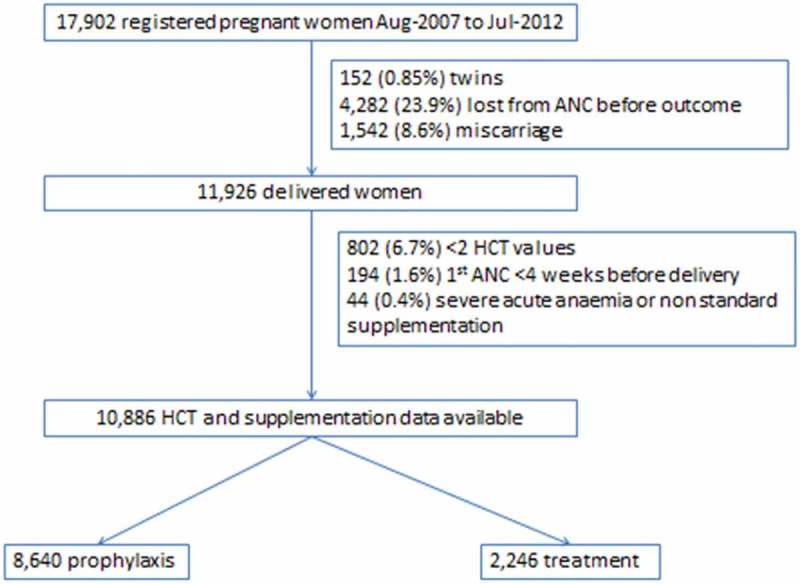
Flow chart.

A total of 60.8% (10,886/17,902) of women registered to ANC had a singleton birth between August 2007 and July 2012 and met the inclusion and exclusion criteria (Figure 1). ANC attendance was high with a median [IQR] of 12 [8–17] visits per woman; just over half (51.2%) presented in the first trimester; and less than 10% of women presented for the first consultation in the third trimester.

In this cohort, 79.4% (8,640/10,886) of women remained on prophylaxis for the duration of pregnancy and 20.6% (2,246) were given anaemia treatment. Women in the prophylaxis group received an average of 7,980 mg of elemental iron during the course of their pregnancies, and women in the treatment group women received an average of 18,060 mg. For women in the treatment group, mild anaemia (91.7%; 2,060/2,246) predominated over moderate anaemia (8.3%, 186/2,246) at the index HCT. The baseline characteristics of these two groups are presented in Table 1. There were 81,158 HCT measurements included, with a median [IQR] of 5 [4–11] measurements per woman. (Table 1).

**Table 1.** Baseline characteristics overall and by haematinic group (prophylaxis or treatment).

		Overall N = 10886	Prophylaxis N = 8640	Treatment N = 2246
Age, years mean ± SD [range]		26 ± 7 [13–50]	26 ± 7 [13–48]	28 ± 7 [14–50]
Gravida, median [IQR; range]		2 [1–4,1–17]	2 [1–4, 1–17]	3 [2–5, 1–14]
Primigravida (G1P0), n (%)		3205 (29.4)	2684 (31.1)	521(23.2)
Trimester first ANC, n (%)	1	5578 (51.2)	4500 (52.1)	1078 (48.0)
	2	4289 (39.4)	3320 (38.4)	969 (43.1)
	3	1019 (9.4)	820 (9.5)	199 (8.9)
Malaria Infection, n (%)		1580 (14.5)	915 (10.6)	665 (29.6)
No. HCT measures, median [IQR; range]		5 [4–11; 2–33]	4 [4–9; 2–33]	11 [8–15; 2–33]
First HCT, mean ±SD [range]		34 ± 4 [12–49]	35 ± 3 [23–49]	31 ± 4 [12–49]
Last HCT, mean ± SD [range]		34 ± 3 [17–49]	34 ± 3 [23–49]	32 ± 3 [17–47]

Abbreviations: ANC antenatal care, EGA estimated gestational age, G gravida, HCT Haematocrit, IQR Inter-quartile range, P parity, SD standard deviation.

^a^Missing data n = 4635. Data are mean ± SD, [range]; or median [IQR; range], or proportion n (%)

**Figure 2.**
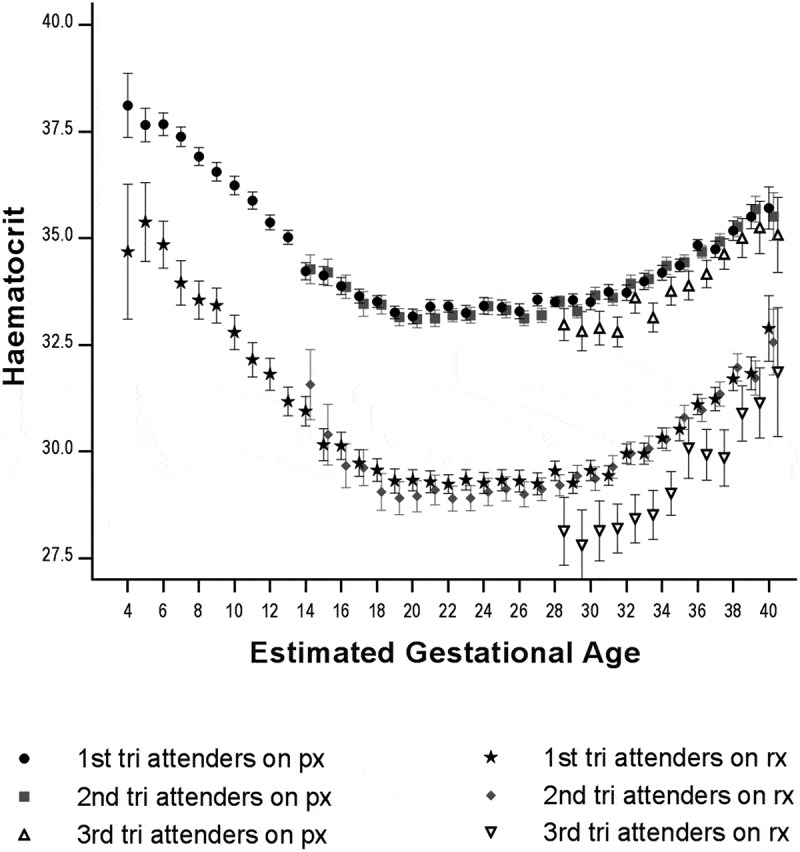
Mean (95%CI) haematocrit according to haematinic group (px – prophylaxis, rx – treatment), gestation (4–40 weeks) and trimester of the first antenatal visit (n = 10,559). N: 1^st^ tri px 4,371; 2^nd^ tri px 3,199; 3^rd^ tri px 811; 1^st^ tri rx 1,039; 2^nd^ tri rx 941; 3^rd^ tri rx 198.Abbreviations: tri, trimester; px, prophylaxis; tx, treatment.

Including pregnancies between 4 and 40 weeks of gestation (n = 10,599), the mean HCT at each gestational age was significantly higher in women under prophylaxis as compared to those under treatment (Figure 2). Women presenting for first ANC in the third trimester of pregnancy had lower mean HCT values in both the prophylactic and treatment groups (Figure 2).

### Timeliness and appropriateness of treatment

Among women who received anaemia treatment, there was a median [IQR, range] of 2 [0–4, 0–31] weeks delay from the first assessed HCT <30% until treatment was commenced, as expected from the protocol (Supplementary file 1). A delay of more than four weeks was experienced by 20.4% (458/2,246). Of those with delayed treatment, 10.5% (48/458) of women proceeded from mild to moderate anaemia and one woman from mild to severe (HCT 25% to 18%) anaemia before treatment was commenced. A record review of these cases identified the main reason for delay as: HCT fluctuation around the 30% cut-point in 49.6% (227/458), prolonged absenteeism before the confirmation HCT was obtained in 28.2% (129/458), human error in 18.8% (86/458) (confirmation HCT obtained but treatment not started), or a positive malaria smear at the confirmation HCT in 3.5% (16/458) in which case treatment was delayed until the blood smear became negative.

An adequate HCT rise of 2% in the first two weeks of treatment was seen in 66.5% (912/1371) of anaemic women where 2-week HCT data was available. The remaining 33.5% (459/1371) of anaemic women were categorized as non-responders, 82.5% (374/459) of whom were offered further investigations. Briefly, IDA, abnormal haemoglobin variants and soil-transmitted helminths were common in this group with refractory anaemia.

Among women who received prophylaxis only, 84.8% (7,326/8,640) had HCT ≥30% at each and every measurement. For the remaining women who had HCT <30%, this was an isolated, single event for most, 77.6% (1,020/1,314). However, in 22.4% (294/1,314) of women a HCT <30% occurred 2 or more times. The majority of the delay for these women (67.0 %, 197/294) involved HCT fluctuation around the 30% cut-point, as permitted by the protocol, with a median of 2 HCT results over the threshold between low HCT results. In the remaining 97 women, the definition of anaemia was met with two or more consecutive HCT <30%, giving a total number of anaemic women of 2,343 (2,246 on treatment and 97 who met the definition but were not treated). Therefore, human error accounted for 4.1% (97/2,343) of anaemic women going untreated.

### Anaemia at delivery

Anaemia was present at delivery in 5.8% (634/10,886) of women: 22.8% (511/2,246) in the treatment group and 1.4% (123/8,640) of the prophylaxis group (p < 0.001).

Risk factors for anaemia at delivery in the treatment group in women who received at least 2 weeks of treatment (n = 2,155) included: not being primigravida, presenting late to ANC (trimester 3), and a delay in starting treatment (>4 weeks); while a higher HCT at diagnosis was protective (Table 2). Malaria in pregnancy was a risk factor for having anaemia and being in the treatment group (Table 1) but did not additionally increase the odds of being anaemic at delivery compared with other women in the treatment group (Table 2).

**Table 2.** Risk factors associated with anaemia at delivery in women in the treatment group who received at least 2 weeks of treatment (n = 2155).

Factor		N 2,155	Delivery HCT <30% n (%)	OR (95% CI)	AOR (95%CI)
Primigravida	Yes	506	78 (15.4)	Reference	Reference
	No	1,649	378 (22.9)	1.63 (1.25, 2.13), p < 0.001	**1.51 (1.14, 2.00), p = 0.004**
T 1^st^ ANC	T 1 or 2	1,976	404 (20.4)	Reference	Reference
	T 3	179	52 (29.1)	1.59 (1.13, 2.24), p = 0.007	**1.46 (1.02, 2.00), p = 0.037**
Malaria in pregnancy	No	1,523	328 (21.5)	Reference	Reference
	Yes	632	128 (20.3)	0.93 (0.74, 1.16), p = 0.507	0.91 (0.71, 1.15), p = 0.415
Delayed treatment	No	1,727	345 (20.0)	Reference	Reference
	Yes, >4 weeks	428	111 (25.9)	1.40 (1.10, 1.79), p = 0.007	**1.56 (1.20, 2.01), p = 0.001**
Mean HCT at diagnosis, %	HCT (%) at start treatment	2,155	n.a.	0.82 (0.78, 0.85), p < 0.001	**0.82 (0.78, 0.85), p < 0.001**

Abbreviations: AOR – Adjusted Odds Ratio; CI – Confidence Interval; ANC antenatal care; HCT – Haematocrit; T trimester.

The normal physiology of pregnancy – whereby haemoconcentration occurs in late pregnancy – reduces the risk of HCT <30% at delivery (Figure 2). The steeper slope of the HCT curve in the treatment group in late pregnancy compared to the prophylaxis group suggests an expected beneficial effect of treatment beyond the normal physiologic process (Figure 2).

## Discussion

Field experience is an important component of guideline development. However, there is a dearth of literature [25] on routine use of guidelines or protocols for detection and treatment of anaemia in pregnancy in remote or low-income settings [26]. This contrasts sharply to the abundance of treatment trials for anaemia in pregnancy [27–29].

Reviews of ANC services from low resource settings usually include summary items relating to anaemia diagnosis and treatment such as the proportion of women tested at least once for anaemia and those who received iron and folate supplements. For example, Osungbade et al. described 390 Nigerian women of whom 42.8% had haemoglobin tested and 36.4% received iron and folate supplements [30]; and a subsequent cohort of 365 women in which 19.2% were tested and 80.3% received supplements [31]. SMRU used the WHO safe motherhood needs assessment to assess these targets with results of 99.5% of women tested and 100% supplemented in 200 randomly chosen delivery records [17]. SMRU’s model of ANC and delivery care includes continuous clinical back-up by doctors, and it is likely this collaborative support for health workers contributes to their success in implementing protocols such as the one described here.

This investigation goes beyond summary indicators to review in detail the clinical use of a protocol in over 10,000 pregnant women. Health-care workers conducted repeated screening during pregnancy attaining a median of five HCT measurements per woman. Human error contributed to the failure of treatment initiation in 4.1% of anaemic women. While human error also contributed to nearly 20% of the cases where there was a delay of more than 4 weeks in starting treatment, prolonged absenteeism from ANC was a bigger challenge, contributing to 28% of the delays.

The main problem with the protocol was the requirement to have two consecutive HCT <30% for confirmation of anaemia. This requirement improved the specificity of HCT as a diagnostic test but unnecessarily complicated the protocol and, most importantly, denied anaemic women prompt treatment. HCT fluctuation around the 30% cut point was common and accounted for half the cases where there was a delay of more than 4 weeks in starting treatment. This delay was associated with a 1.5 fold higher odds of anaemia at delivery. Requiring a second HCT was also problematic in women who had one HCT <30% before a period of absenteeism. This evaluation has prompted a protocol change and HCT confirmation is now omitted, potentially increasing the number of women treated unnecessarily but impacting on missed opportunities for treatment and on anaemia at delivery.

The gestational effect on HCT has been described previously in this setting [32] and in the international literature [33] and is reassuring in terms of robustness of the data. Most women were started on prophylactic haematinics (low dose iron and folate) and did not become anaemic during the pregnancy, receiving a median of 20 weeks of prophylaxis. Though causality cannot be proven in this study, women in the prophylactic group who presented in trimesters 1 and 2 had higher mean HCT per gestational age week compared to women presenting late, in trimester 3 (Figure 2). Decreased rates of anaemia in early ANC attenders are congruent with other benefits of early ANC care such as accurate gestational age determination, and nutritional counselling [34].

The main limitation of this analysis is that as all women received prophylactic haematinics, it cannot be determined from this dataset how many cases of anaemia were prevented by high uptake of prophylaxis or what the population prevalence of anaemia in pregnancy would be without this intervention. While two-thirds of anaemic women responded to treatment doses of iron and folate in the short term, the true prevalence of IDA and other causes of anaemia (such as inherited red blood cell disorders) [12] remains to be characterized in this population.

## Conclusions

Health workers, with appropriate clinical support, effectively managed screening of anaemia by routine measurement of HCT and diagnosed and treated >95% of anaemic patients. An important outcome of this evaluation was that the clinical definition of HCT was changed to diminish missed opportunities for initiating treatment doses of haematinics. A simplified protocol initiating treatment at the first opportunity has the potential to significantly reduce anaemia at delivery.

## Paper context

Anaemia in pregnancy is common with detrimental effects to both mother and fetus from iron deficiency. Protocol evaluation found high health worker compliance but the requirement of two consecutive low HCT measurements to diagnose anaemia led to missed opportunities for treatment. Four percent anaemic women missed treatment due to human error. These findings support the use of a simple protocol and have changed local practice to commence treatment at the first low HCT.

## References

[1] The World Health Organization The global prevalence of anaemia in 2011. Geneva: The World Health Organization; 2015.

[2] Filippi V, Chou D, Ronsmans C, et al Levels and causes of maternal mortality and morbidity In: Black RE, Laxminarayan R, Temmerman M, et al, editors. Reproductive, maternal, newborn, and child health: disease control priorities. 3rd ed. Vol. 2 Washington (DC): The International Bank for Reconstruction and Development/The World Bank; 2016:51–70.

[3] Khan KS, Wojdyla D, Say L, et al WHO analysis of causes of maternal death: a systematic review. Lancet. 2006;367:1066–8. 16581405 10.1016/S0140-6736(06)68397-9

[4] Bhutta ZA, Imdad A, Ramakrishnan U, et al Is it time to replace iron folate supplements in pregnancy with multiple micronutrients? Paediatr Perinat Epidemiol. 2012;26 Suppl 1:27–35. 10.1111/j.1365-3016.2012.01313.x 22742600

[5] The World Health Organization WHO recommendations on antenatal care for a positive pregnancy experience. Geneva: World Health Organization; 2016 p. 152. 28079998

[6] Radlowski EC, Johnson RW. Perinatal iron deficiency and neurocognitive development. Front Hum Neurosci. 2013;7 DOI:10.3389/fnhum.2013.00585 PMC3779843 24065908

[7] Sanghvi TG, Harvey PWJ, Wainwright E Maternal iron-folic acid supplementation programs: evidence of impact and implementation. Food Nutr Bull. 2010;31:S100–S107. 20715594 10.1177/15648265100312S202

[8] Winichagoon P Prevention and control of anemia: Thailand experiences. J Nutr. 2002;132:862S–866S. 11925499 10.1093/jn/132.4.862S

[9] Nosten F, Ter Kuile F, Maelankirri L, et al Malaria during pregnancy in an area of unstable endemicity. Trans R Soc Trop Med Hyg. 1991;85:424–429. 1836685 10.1016/0035-9203(91)90205-d

[10] Boel M, Carrara VI, Rijken M, et al Complex interactions between soil-transmitted helminths and malaria in pregnant women on the thai-burmese border. PLoS Negl Trop Dis. 2010;4:e887. 21103367 10.1371/journal.pntd.0000887 PMC2982827

[11] McGready R, Boel M, Rijken MJ, et al Effect of early detection and treatment on malaria related maternal mortality on the north-western border of Thailand 1986-2010. PloS One. 2012;7:e40244. 22815732 10.1371/journal.pone.0040244 PMC3399834

[12] Bancone G, Gilder ME, Chowwiwat N, et al Prevalences of inherited red blood cell disorders in pregnant women of different ethnicities living along the Thailand-Myanmar border. Wellcome Open Res. 2017;2:72. 29181452 10.12688/wellcomeopenres.12338.2 PMC5686509

[13] Spector JM, Agrawal P, Kodkany B, et al Improving quality of care for maternal and newborn health: prospective pilot study of the WHO safe childbirth checklist program. PloS One. 2012;7:e35151. 22615733 10.1371/journal.pone.0035151 PMC3353951

[14] Yip R Iron supplementation during pregnancy: is it effective? Am J Clin Nutr. 1996;63:853–855. 8644677 10.1093/ajcn/63.6.853

[15] Jaroensuk J, Stoesser N, Leimanis ML, et al Treatment of suspected hyper-reactive malarial splenomegaly (HMS) in pregnancy with mefloquine. Am J Trop Med Hyg. 2014;90:609–611. 24591439 10.4269/ajtmh.13-0706 PMC3973501

[16] Rijken MJ Malaria in pregnancy: ultrasound studies of fetal growth [PhD]. Utrecht (Netherlands): Utrecht University; 2012.

[17] Hoogenboom G, Thwin MM, Velink K, et al Quality of intrapartum care by skilled birth attendants in a refugee clinic on the Thai-Myanmar border: a survey using WHO safe motherhood needs assessment. BMC Pregnancy Childbirth. 2015;15 DOI:10.1186/s12884-015-0444-0 PMC4332741 25652646

[18] White AL, Min TH, Gross MM, et al Accelerated training of skilled birth attendants in a marginalized population on the Thai-Myanmar border: a multiple methods program evaluation. PloS One. 2016;11:e0164363. 27711144 10.1371/journal.pone.0164363 PMC5053505

[19] Rijken MJ, Mulder EJH, Papageorghiou AT, et al Quality of ultrasound biometry obtained by local health workers in a refugee camp on the Thai-Burmese border. Ultrasound Obstet Gynecol. 2012;40:151–157. 22262286 10.1002/uog.11091 PMC3443371

[20] McGready R, Simpson JA, Cho T, et al Postpartum thiamine deficiency in a Karen displaced population. Am J Clin Nutr. 2001;74:808–813. 11722964 10.1093/ajcn/74.6.808

[21] Lee SJ, Stepniewska K, Anstey N, et al The relationship between the haemoglobin concentration and the haematocrit in Plasmodium falciparum malaria. Malar J. 2008;7:149. 18673575 10.1186/1475-2875-7-149 PMC2515851

[22] Pasricha S-R, Colman K, Centeno-Tablante E, et al Revisiting WHO haemoglobin thresholds to define anaemia in clinical medicine and public health. Lancet Haematol. 2018;5:e60–e62. 29406148 10.1016/S2352-3026(18)30004-8

[23] Thirup P Haematocrit: within-subject and seasonal variation. Sports Med. 2003;33:231–243. 12656642 10.2165/00007256-200333030-00005

[24] Daru J, Allotey J, Peña-Rosas JP, et al Serum ferritin thresholds for the diagnosis of iron deficiency in pregnancy: a systematic review. Transfus Med. 2017;27:167–174. 28425182 10.1111/tme.12408 PMC5763396

[25] Stoltzfus RJ, Pillai G Measuring performance: a strategy to improve programs. J Nutr. 2002;132:845S–848S. 11925494 10.1093/jn/132.4.845S

[26] Parsons JE, Merlin TL, Taylor JE, et al Evidence-based practice in rural and remote clinical practice: where is the evidence? Aust J Rural Health. 2003;11:242–248. 14641222

[27] Goonewardene IMR, Senadheera DI Randomized control trial comparing effectiveness of weekly versus daily antenatal oral iron supplementation in preventing anemia during pregnancy: oral iron to prevent anemia in pregnancy. J Obstetrics Gynaecol Res. 2018;44:417–424. 10.1111/jog.13546 29271022

[28] Naqash A, Ara R, Bader GN Effectiveness and safety of ferric carboxymaltose compared to iron sucrose in women with iron deficiency anemia: phase IV clinical trials. BMC Women’s Health. 2018;18 DOI:10.1186/s12905-017-0506-8 PMC5755312 29304848

[29] Bah A, Wegmuller R, Cerami C, et al A double blind randomised controlled trial comparing standard dose of iron supplementation for pregnant women with two screen-and-treat approaches using hepcidin as a biomarker for ready and safe to receive iron. BMC Pregnancy Childbirth. 2016;16 DOI:10.1186/s12884-016-0934-8 PMC4944263 27411564

[30] Osungbade K, Oginni S, Olumide A Content of antenatal care services in secondary health care facilities in Nigeria: implication for quality of maternal health care. Int J Qual Health Care. 2008;20:346–351. 18621778 10.1093/intqhc/mzn026

[31] Osungbade KO, Shaahu VN, Uchendu OC Clinical audit of antenatal service provision in Nigeria. Health Care Women Int. 2011;32:441–452. 21476162 10.1080/07399332.2010.517878

[32] Nosten F, McGready R, Simpson JA, et al Effects of *Plasmodium vivax* malaria in pregnancy. Lancet. 1999;354:546–549. 10470698 10.1016/s0140-6736(98)09247-2

[33] Taylor DJ, Lind T Red cell mass during and after normal pregnancy. Br J Obstet Gynaecol. 1979;86:364–370. 465384 10.1111/j.1471-0528.1979.tb10611.x

[34] Moller A-B, Petzold M, Chou D, et al Early antenatal care visit: a systematic analysis of regional and global levels and trends of coverage from 1990 to 2013. Lancet Glob Health. 2017;5:e977–e983. 28911763 10.1016/S2214-109X(17)30325-X PMC5603717

