# Supplementary figures and images for: Evaluation of a treatment protocol for anaemia in pregnancy nested in routine antenatal care in a limited-resource setting
Source: Glob Health Action. 2019 Jun 17;12(1):1621589. doi: 10.1080/16549716.2019.1621589 (PMC6586122; doi:10.1080/16549716.2019.1621589)

**Supplementary File Figure. Anaemia treatment protocol**

**
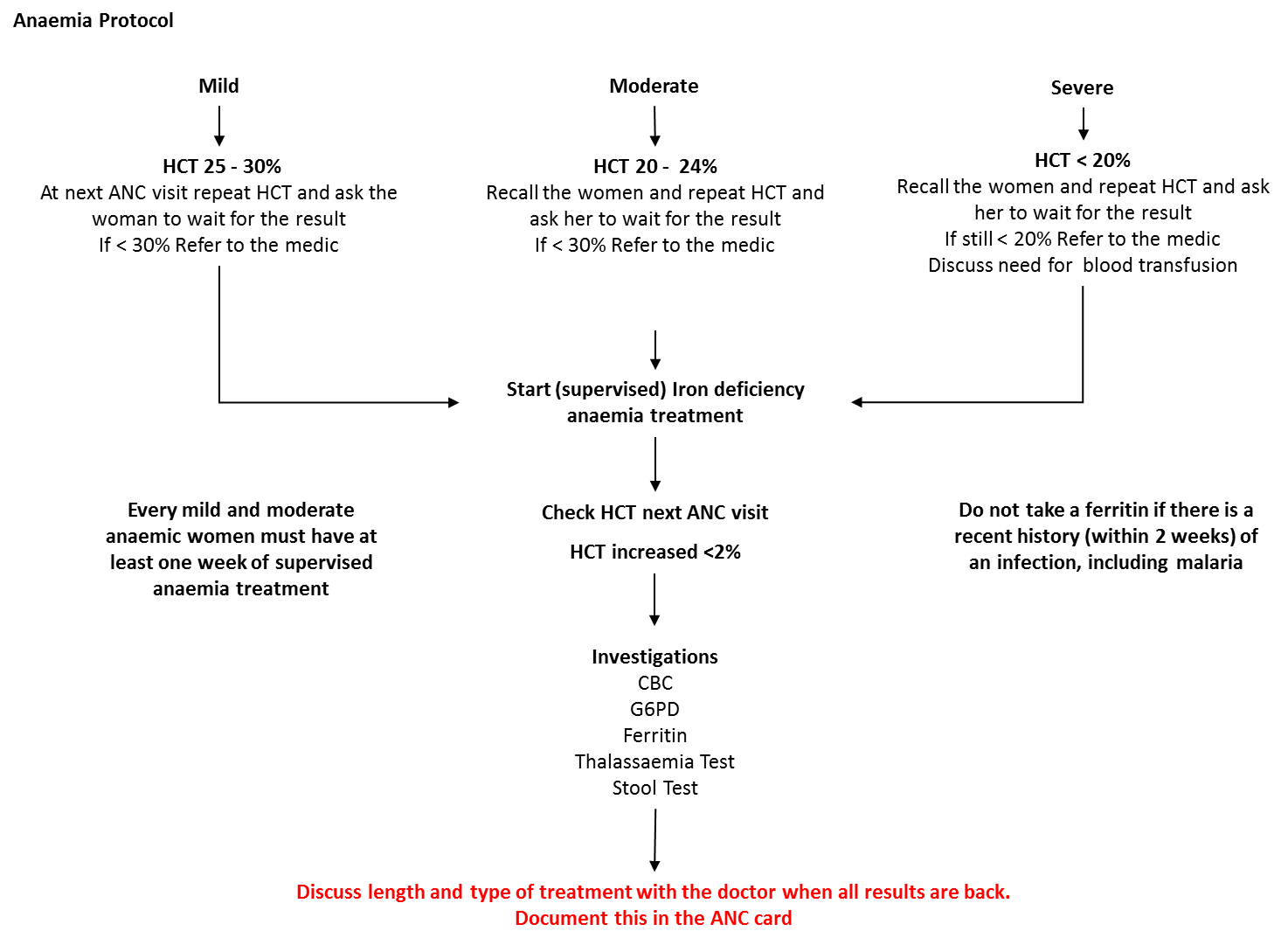
**

Supplement: Supplemental Material [file ZGHA_A_1621589_SM9686.docx]
